# Identification of Wheat Inflorescence Development-Related Genes Using a Comparative Transcriptomics Approach

**DOI:** 10.1155/2018/6897032

**Published:** 2018-02-08

**Authors:** Lingjie Ma, Sheng-Wei Ma, Qingyan Deng, Yang Yuan, Zhaoyan Wei, Haiyan Jia, Zhengqiang Ma

**Affiliations:** The Applied Plant Genomics Laboratory of Crop Genomics and Bioinformatics Centre, Nanjing Agricultural University, Jiangsu 210095, China

## Abstract

Inflorescence represents the highly specialized plant tissue producing the grains. Although key genes regulating flower initiation and development are conserved, the mechanism regulating fertility is still not well explained. To identify genes and gene network underlying inflorescence morphology and fertility of bread wheat, expressed sequence tags (ESTs) from different tissues were analyzed using a comparative transcriptomics approach. Based on statistical comparison of EST frequencies of individual genes in EST pools representing different tissues and verification with RT-PCR and RNA-seq data, 170 genes of 59 gene sets predominantly expressed in the inflorescence were obtained. Nearly one-third of the gene sets displayed differentiated expression profiles in terms of their subgenome orthologs. The identified genes, most of which were predominantly expressed in anthers, encode proteins involved in wheat floral identity determination, anther and pollen development, pollen-pistil interaction, and others. Particularly, 25 annotated gene sets are associated with pollen wall formation, of which 18 encode enzymes or proteins participating in lipid metabolic pathway, including fatty acid *ω*-hydroxylation, alkane and fatty alcohol biosynthesis, and glycerophospholipid metabolism. We showed that the comparative transcriptomics approach was effective in identifying genes for reproductive development and found that lipid metabolism was particularly active in wheat anthers.

## 1. Introduction

Inflorescences are the reproductive architectures of plants, composed of stems, stalks, bracts, and flowers. *Poaceae* (also called *Gramineae*) is one of the largest families in the monocotyledonous flowering plants, including the major cereal crops, such as maize (*Zea mays* L.), rice (*Oryza sativa* L.), and wheat (*Triticum aestivum* L.). Inflorescences of this family are characterized by their panicle or spike shapes [1], complex branches, and unique spikelets, as well as inconspicuous and anemophilous flowers without obvious petals and sepals [2, 3]. Species in the genus *Triticum* takes the shape of the spike with the spikelets, each containing one or more florets, attached to rachis nodes. The wheat floret consists of a carpel with its ovary, style, and stigma, with three anthers attached to the base through slender filaments, which are enclosed by bract-like organs called lemma and palea.

Inflorescence development and regulation have attracted great attention from plant biologists and crop breeders since they are crucial for reproduction of flowering plants and for production of food grains in cereal crops. Although the key genes regulating the flower initiation and development are conserved in higher plants [4, 5], the diversity of reproductive structure and behaviors is still not well explained. Transcriptomes reflect the complete set of RNA transcripts expressed by the genome under developmentally or physiologically distinct states; therefore, its comparison allows identification of genes under common regulation. High-throughput methods, such as serial analysis of gene expression (SAGE) [6], microarray technology [7], and next-generation sequencing [8] have enabled transcriptome studies in an unprecedented scale in many plant species, especially in those with full genome sequence information available. This has led to the discovery of a number of genes involved in flower development. These genes display tempo-spatial expression patterns not only in transcriptome level [9–12] but also at the proteome level, such as those in pollen development of tomatoes [13], indicating their strictly regulated functions. Anther tissues were used in most of these studies because they are easy to isolate and specific in biological roles. The availability of these data has shed some light on the gene networks that contribute to the formation of unique reproductive structures and flower development, although it might still not be enough because of the particularity of different plant species and highly specific flower components.

In bread wheat (*T. aestivum* L.), an allohexaploid species (2*n* = 42) with three closely related subgenomes, that is, A, B, and D [14], large-scale transcriptomic investigations have been conducted in some tissue types. Crismani et al. performed microarray-based expression analysis of anthers across various stages of meiosis in an attempt to build the link between the model and wheat and identify early meiotic genes [15]. McIntosh et al. investigated the transcriptome in wheat caryopsis development using data generated by SAGE [16]. Yang et al. compared the RNA-seq tags of pistillody stamen and pistil from a pistillody wheat mutant and stamen from the wild-type control to identify differentially expressed genes [17]. In these studies, the annotation of the identified transcripts was based on short tags or sequence reads which is impossible to ensure accuracy for species such as wheat that has a complex genome and does not have detailed sequence information so far. Moreover, gene identity determination according to short sequence fragments could be problematic due to functional diversification of the homoeologous genes in polyploidy species.

Expressed sequence tags (ESTs) are single-pass sequence reads by sequencing cDNA libraries and usually have >400-base read length. They represent part of the transcriptome in a given tissue and/or at a given developmental stage. As of January 1, 2013, over one million ESTs are available for wheat in GenBank of the National Center for Biotechnology Information (NCBI) and are valuable for identifying genes involved in biotic and abiotic stress [18–22], kernel development and quality [23, 24], and development [21, 25, 26].

In this paper, we reported mining of 170 genes predominantly expressed in floral organs through comparative transcriptomic profiling using ESTs of 67 wheat cDNA libraries deposited in GenBank genes and the identification of a few metabolic pathways involved in anther development.

## 2. Materials and Methods

### 2.1. Wheat ESTs and Contigs

ESTs were downloaded from the NCBI dbEST database (ftp://ncbi.nlm.nih.gov/repository/dbEST). The ESTs used in this study were produced with 67 cDNA libraries prepared using inflorescences (spike at flowering or before flowering, anther, pistil, ovary, palea, and lemma), roots, stems, leaves (including seedlings and crown tissues), and seeds (matured or immature embryos) from normally grown seedlings or plants and represented 434,658 cloning events (Table S1). Libraries subjected to enrichment or normalization treatment and those with less than 1000 ESTs were not included in the expression profiling analysis. The 163rd release of unique wheat transcripts, including 77,657 contigs, was downloaded from the PUT database (http://www.plantgdb.org).

### 2.2. Gene Mining

To identify putative genes predominantly expressed in wheat flowers, BLAST search was conducted using the PUT contig sequences as queries against the EST database consisting of the 67 cDNA libraries. ESTs matching each PUT contig with ≥90% homology were recorded and classified according to their library origins from the five aforementioned tissue types. The contigs with matched ESTs solely from inflorescences and those with significantly more matched ESTs from inflorescence tissues than from any of the other four types of tissues were considered to be the putative genes predominantly expressed in inflorescences. The probability to achieve the ESTs matching to a contig in the noninflorescence libraries was estimated based on the random sampling principle using the equation: *P* = *C*_*N*_^*n*^ *f* ^*n*^(1 − *f*)^*N*−*n*^, as described by Ding et al. [27], where *f* is the EST frequency of a contig in the inflorescence libraries estimated by dividing the number of matched ESTs by the total number of ESTs in the libraries and *n* and *N* are the number of matched ESTs and the total number of ESTs in libraries of other tissues types, respectively. A sampling probability ≤ 0.0001 was considered an indication of significant difference in expression levels between the inflorescence tissues and noninflorescence tissues.

Chromosome-specific genomic DNA sequences corresponding to the retained contigs were obtained by retrieving the chromosome-assigned homologous scaffolds in the TGAC database [28] (http://www.tgac.ac.uk/grassroots-genomics) that showed 95% homology to the contig sequences. Coding DNA sequences (CDS) corresponding to the contigs in the scaffolds were predicted using the gene-finding program FGENESH [29] (http://linux1.softberry.com) and verified by alignment with the homologous ESTs. Scaffolds that did not contain the full-length target CDS were extended via in silico walking, using a parameter of 100% match, with the Roche 454 sequence reads of Chinese Spring [30] (http://www.cerealsdb.uk.net).

To confirm the expression predominance of the identified genes in wheat flowers, ESTs with ≥99% homology to the individual CDS identified from the sixty-seven libraries were subjected to further analysis. The sampling probability to achieve the EST frequency of a CDS in the noninflorescence libraries was again calculated. A sampling probability ≤ 0.01 was used as the threshold for declaration of significant difference. Expression specificity of the candidate genes was estimated similarly.

The identified genes were coded in numerical order with the prefix “IDG” (inflorescence development-related gene). Orthologous genes were given a common name but marked with a chromosome assignment suffix. Multiple copies derived from duplication of an ancestral gene at the same chromosome were differentiated by a numerical suffix.

### 2.3. RNA-Seq Data Analysis

RNA-data sets used in the analysis included the developmental time course series in five tissues (spike or inflorescence, root, leaf, grain, and stem) each with three different developmental stages. The stages of inflorescence included Z32, two nodes stage; Z39, meiosis; Z65, anthesis of C.S. [31] (http://wheat.pw.usda.gov/WheatExp), those for C.S. pistil and stamen by Yang et al. [17], and those for anther at meiosis data from the URGI public database (https://wheat-urgi.versailles.inra.fr/Seq-Repository/Expression). After trimmed adapters and removing vectors and low quality reads with AdapterRemoval in a setting of quality base = 33 [32], the RNA-seq reads were mapped to the sequence database consisting of genomic DNA sequences of each identified candidate gene using HISAT2 [33]. FPKM of a gene was estimated with reads matching to each gene with 100% identity, which were counted using featureCounts with both readExtension5 and readExtension3 set at 70 [34].

To estimate the relative expression specificity (RES) of a gene in a certain tissue (A), the tissue (B) in which the gene was most highly expressed among all but tissue A was identified. RES was then measured by dividing the difference of the normalized expression values between A and B by the expression value in A. The higher the RES value is, the more specific the gene expression is in A. Significance of the expression difference between two tissues was examined via *χ* ^2^ test. A gene was considered to be differentially expressed when the difference reached significance at *P* = 0.05.

To reflect the relative expression abundance of each gene in each tissue among the identified genes, a heat map was drawn with the quotients obtained from dividing the FPKM value (the maximal one if two or more developmental stages of a tissue were involved) of a specific gene in a tissue by the FPKM means of all genes. A log-transformation was applied to facilitate the mapping drawing.

### 2.4. RT-PCR

Root, node, internode, flag leaf, glume, lemma, palea, lodicule, stamen, pistil, and rachis tissues of the common wheat landrace “Wangshuibai,” grown in a field during the normal growing season, were collected at the heading stage, and developing kernels were collected at the 9th day postanthesis for RNA extraction. RNA was extracted using TRIzol reagent (Invitrogen, CA) following the manufacturer's protocol and quantified with an Ultrospec 2100 Pro spectrometer (Amersham Pharmacia, UK). To eliminate DNA contamination, RNA samples were treated with RNase-free DNase I (Fermentas, Canada), following the product manual. First-strand cDNA was synthesized using oligo(dT) primer with 3 *μ*g of total RNA using M-MLV reverse transcriptase (Life Technologies, CA) according to the manufacturer's instructions.

Semiquantitative RT-PCR (sqRT-PCR) was performed in a 25 *μ*l total reaction volume supplemented with 10–20 ng of first-strand cDNA as the template, 5 pmol concentration of each primer, 5 nmol dNTPs for each, 1 U rTaq DNA polymerase (Takara, Japan), and 1x PCR buffer supplied together with the enzyme. The wheat *α*-tubulin gene was used in calibrating cDNA templates. The following thermal cycle profile was observed: 94°C for 3 min; 26–32 cycles of 94°C for 20 s, 56–62°C (depending on the primer sets) for 25 s, and 72°C for 30 s, and a final extension step of 72°C for 5 min. PCR products were resolved in 2.0% (*w*/*v*) agarose gels and visualized with ethidium bromide staining. RT-PCR reactions were independently repeated three times or more to ensure reproducibility.

Quantitative real-time RT-PCR (qRT-PCR) amplifications in 20 *μ*L volumes, containing 10 *μ*L SYBRÒ Green qRCR Mix (Toyoba), 20 ng template, and 8 pmol of each primer, were performed using the StepOneTM Real-Time PCR instrument (Applied Biosystems), following the protocol described in [35]. The reactions were conducted in triplicate. The cycle threshold values for each target gene were normalized based on values obtained in corresponding reactions for the wheat *α*-tubulin gene. The relative expression was estimated by employing the 2^−∆∆CT^ method [36].

The RT-PCR primers used in the present study were designed with MacVector 11 (MacVector, NC) and are listed in Table S2. The product length ranged from 150–300 bp.

### 2.5. Gene Annotation and Pathway Assignment

Genes were functionally annotated through homologous search of the NCBI nonredundant (Nr) protein, KEGG [37] (http://www.kegg.jp/blastkoala), and Pfam [38] (http://pfam.xfam.org) databases. Subcellular locations of the proteins were predicted using the TargetP 1.1 server [39] (http://www.cbs.dtu.dk/services/TargetP). Pathway assignments were based on KEGG pathway mapping (http://www.kegg.jp/kegg/tool/map_pathway1.html) and keyword search of the plant metabolic pathway databases (http://www.plantcyc.org).

## 3. Results

### 3.1. Identification of Genes Preferentially Expressed in Inflorescence

Of the 67 cDNA libraries in line with the screening conditions, 29 libraries containing 140,092 sequences were from seed tissues; only three cDNA libraries including 17,732 sequences were from stem tissues (Table S1). The number of ESTs in each library ranged from 1000 to more than 10,000. For identification of the inflorescence development-related genes, these libraries were classified into five types according to the tissues used in library preparation, including seedling-stage leaf and stem, seedling to tillering-stage root, seed (from DPA3 to mature), and inflorescence (including premeiotic anthers, anthers at meiosis, pistil and ovary, immature inflorescence, lemma and palea, spike before flowering, and spike at flowering).

Alignment of the 77,657 wheat EST contigs, downloaded from the PlantGDB-assembled unique transcript (PUT) database, with ESTs of the abovementioned cDNA libraries led to the identification of 335 contigs that matched (95% homology) significantly more ESTs from inflorescence tissues, including nearly one-third with ESTs only from inflorescence tissue libraries. Using these contig sequences as queries in search of the TGAC database [28] with 90% as the homology threshold, 318 nonabundant genomic DNA sequences were obtained, 294 of which yield the expected open reading frames (ORF) with EST support in gene prediction with the gene-finding program FGENESH [29]. With 99% homology as the cutoff, 187 genes matched significantly more ESTs from the inflorescence tissues than from any of the other four types of tissues (*P* = 0.01). We disregarded 17 of these genes that were neither more abundant in the inflorescence RNA-seq datasets nor in the anther and pistil data sets. Interestingly, most ESTs matching to 14 of the 17 genes came from Ogihara's unpublished cDNA libraries Wh_FL or Wh_f, which were constructed with spikelets or spikes at flowering stage. Probably, these genes are expressed at a developmental inflorescence stage not included in the tissues for the RNA sequencing. The remaining 170 genes represented 59 inflorescence development-related nonredundant gene sets, since wheat is an allohexaploid species and the majority of the genes have three orthologous copies (Table 1 and Table S3).

In terms of genomic distribution, the identified genes distributed to chromosomes of homoeologous groups 1, 3, and 6 accounted for 68.2%, those to group 4 chromosomes accounted for only 5.3%. Majority of the nonredundant gene sets have homologs in all three homoeologous chromosomes; however, not all of them showed inflorescence development-related preferential expression, suggesting functional differentiation have occurred among them. Of the 59 nonredundant gene sets, 13 had inflorescence development-related homologs in only two of three subgenomes, eight were solely identified in a single subgenome. Eight nonredundant gene sets had intrachromosomal duplications, of which five were distributed to the homoeologous group 6 chromosomes. A notable example was *IDG042* that had duplications in all three group 6 chromosomes, with a total of 14 copies showing inflorescence development-related preferential expression.

### 3.2. Expression Specificity of the Identified Genes

**Figure 1:**
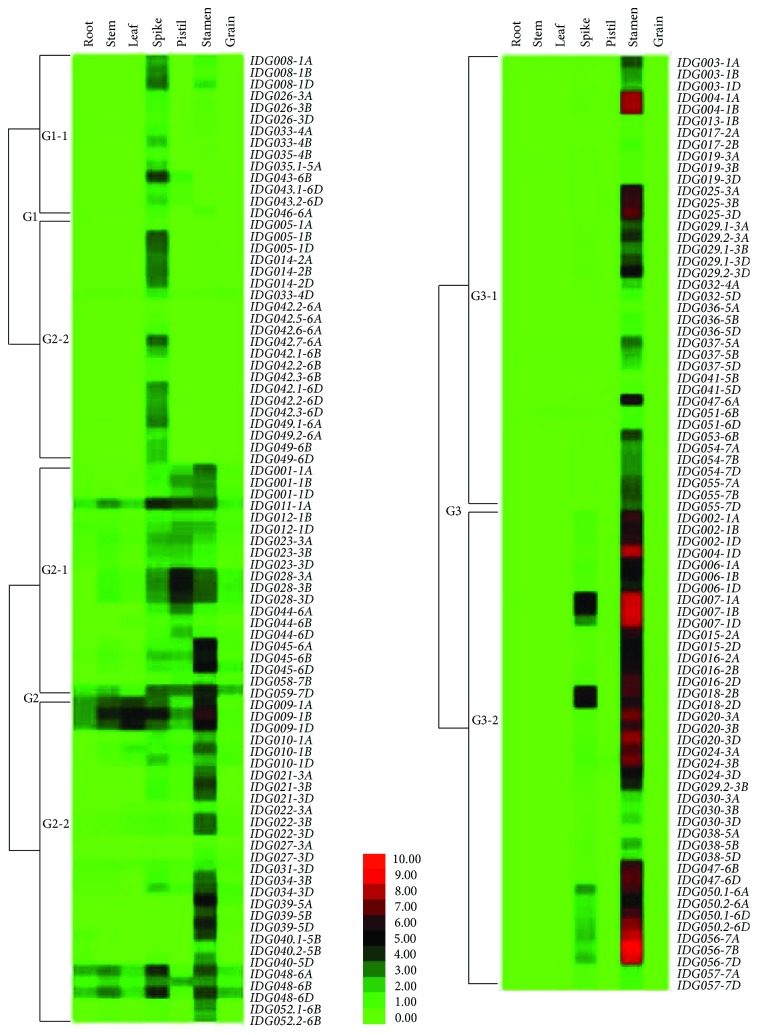
Relative expression abundance of the identified genes in different tissues based on RNA-seq data. Apart from seven genes with a negligible number of reads, the remaining 163 genes were divided according to their expression profiles into three groups, each of which had two subgroups.

In mining genes predominantly expressed in inflorescence, we considered all tissues from inflorescence as a whole. It was noted, in the EST analysis, that some genes were solely expressed in spikes at or before anthesis, some were solely in stamen or in pistil, apart from those expressed more abundantly in spikes than in other tissues. To validate the expression patterns of these genes, we tested the significance of expression difference and estimated the expression specificity (RES) of inflorescence (including the stages of Z32, Z39, and Z65), stamen, and pistil relative to the individual vegetative tissues (including kernel) using the RNA-seq data. Based on the expression profiles, we were able to classify the 170 genes into three groups, each with two subgroups (Figure 1 and Table S3). Within each subgroup, the expression profiles were similar between genes, but the relative abundances were not identical even between orthologous genes.

The first group, G1, consisted of 42 genes from 10 gene sets. Overall, they had a low expression level and were expressed more abundantly and specifically in majority of the cases, in the inflorescence as a whole. A few genes, such as *IDG035.2-5A*, *IDG035.1-4D*, *IDG035.2-4D*, *IDG042.1-6A*, *IDG042.3-6A*, *IDG042.4-6A*, and *IDG042.8-6A*, were supported by ESTs but were matched to a negligible number of reads in the RNA-seq datasets. They were classified together with their orthologous or paralogous homologs, since their expression profiles in EST analysis were similar. Genes in subgroup G1-1 also showed enhanced expression, even though less abundantly, in stamen or pistil. Different from those in G1-1, genes in subgroup G1-2 had negligible fragments per kilobase of cDNA model per million mapped reads (FPKM) values from tissues other than the inflorescence.

The second group, G2, consisted of 20 gene sets and 48 genes. This group was basically characterized by expression in one or more of the vegetative tissues and an even higher level of expression in the stamen and/or pistil. *IDG011-1A* was the only exception, which was predominantly expressed in the inflorescence as a whole in spite of a significantly higher expression in the stamen and pistil relative to the vegetative tissues. Genes in subgroup G2-1 all had a significantly higher level of expression in the pistil relative to the vegetative tissues, and except for *IDG044-6B* and *IDG044-6D*, a significantly higher level of expression in the stamen as well. A few genes in this subgroup were expressed more abundantly in the inflorescence (*IDG011-1A*) or in the stamen (*IDG001-1A*, *IDG001-1D*, and *IDG045*). Genes in subgroup G2-2 were expressed more abundantly in the stamen relative to the vegetative tissues, but their expression levels in the pistil were not different from or even lower than those in at least one of the vegetative tissues.

Group G3 was the largest group, including 80 genes from 29 gene sets, characterized by a predominant expression in stamen and a negligible level of expression in the vegetative tissues. The RES values (stamen versus vegetative tissues) were high, ranged from 0.82–1.0. G3-1 consisted of 39 genes, all with a negligible FPKM value from the inflorescence. The remaining genes were different from genes in G3-1 by a significantly enhanced expression in the inflorescence as well relative to the vegetative tissues, although the expression level was much lower in most cases.

**Figure 2:**
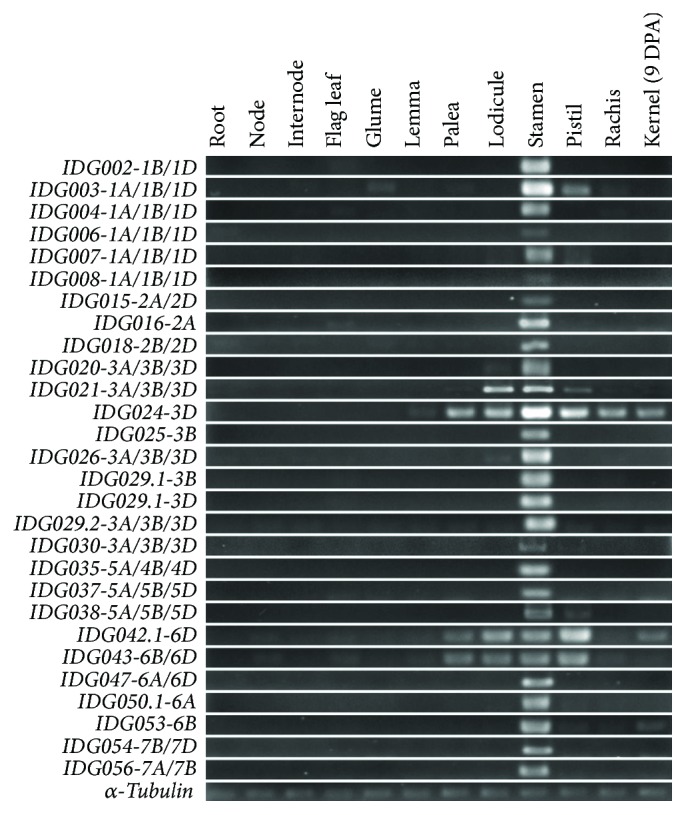
Expression of a selected set of identified genes in different tissues. The tissues used included kernel 9th day postanthesis, root, node, internode, flag leaf, glume, lemma, palea, lodicule, stamen, pistil, and rachis at the heading stage of common wheat landrace “Wangshuibai.”

To verify the expression specificity experimentally, sqRT-PCR was performed with tissues of root, node, internode, flag leaf, glume, lemma, palea, lodicule, stamen, pistil, rachis, and kernel 9th day postanthesis, using 27 pairs of primers that corresponds to 64 members of the identified genes (Table S2). All PCR reactions revealed a pattern of predominant expression in at least one of the floral tissues or organs but not in kernels and vegetative organs (Figure 2), which, by and large, were in agreement with results from the EST and RNA-seq data analysis. The qRT-PCR of a selected set of genes, including those with a relatively lower expression level and those expressed in multiple tissues, further confirmed these results (Figure S1).

Generally speaking, the tissue expression profiles between orthologous genes inferred based on the EST data were similar to those from the RNA-seq analysis. But a few exceptions were noted. In most of these cases, a low expression level of certain orthologous members was likely the cause of the discrepancy, for instance, some members of *IDG035* and *IDG042*.

### 3.3. Functional Annotation of the Identified Genes

Forty-nine of the identified gene sets were annotated via homology search and classified into five categories according to their putative biological functions (Table 2). It has to be mentioned that some genes could functionally fall into multiple categories.

The first category included only two gene sets; both encode allergenic proteins. The biological functions of this class of proteins in floral development have not been well characterized. *IDG035* encodes group 3 grass pollen allergens, which have sequence similarity to expansins that promote plant cell wall enlargement and thereby serve as cell wall-loosening agents [40].

The second category included 11 gene sets, most of which belonged to the G2 expression type. In this category, five gene sets code for proteins related to JA, ET, and GA signaling, three for MADS-box transcription factors and three for H2A and H2B proteins. The MADS-box transcription factor proteins encoded by *IDG001* and *IDG021* have 87% similarity. *IDG001* is orthologous to the rice *OsMADS4*. According to the ABCDE model for floral organ identity specification [41], *IDG001* and *IDG021* belong to class B MADS-box genes. *IDG044* encodes an AGL6-like MADS transcription factor and is functionally similar to class E genes [42].

The third category included 28 gene sets, accounting for 57% of the annotated. They were all predominantly or specifically expressed in stamen. Most of them were associated with substance production, transportation, and assembly for anther and pollen development. Of this category, 18 gene sets code for proteins associated with fatty acid and lipid metabolism. Homologs in other plants of most of these genes have been associated with the process of pollen wall development, such as suberin biosynthesis [43, 44], cutin biosynthesis [45–47], pollen sporopollenin biosynthesis [48], and pollen exine formation [49–51]. Other genes in this category have also been associated with anther and pollen development. *IDG038* encodes an acidic peroxidase that might participate in the synthesis of phenylpropanoids present in sporopollenin [52]. The products encoded by *IDG007*, *IDG018*, *IDG050*, *IDG055*, and *IDG056* were related to pollen wall formation [53–55]. Both *IDG004* and *IDG025* code for anther-specific RTS-like proteins, required for male fertility and affecting tapetal development [56]. *IDG006* encodes a galactosyltransferase, which is implicated in the accumulation control of glycosylated flavonols in pollen [57]. In *Arabidopsis*, a type II *β*-(1,3)-galactosyltransferase is required for pollen exine development [58]. *IDG020* codes for a late cornified envelope- (LCE-) like proline-rich protein. LCE proteins are involved in the cornified cell envelope assembly of skins and associated with ROS detoxification [59].

The fourth category only has four gene sets and is associated with pollination and pollen-stigma interactions. For instance, the subtilisin-like protease encoded by *IDG002* was related to anther dehiscence [60]; the products of *IDG008* were related to pollen tube growth [61]; the chemocyanins encoded by *IDG040* could be involved in the pollination process [62] and induce pollen tube chemotropism as a diffusible chemotropic factor [63]. *IDG028* codes for a protodermal factor 1-like protein mainly in the pistil. This protein is related to reactive oxygen species (ROS) homeostasis [64]. ROS are involved in pollen tube growth and rupture [65, 66], implying the role of modulating ROS levels in male reproductive development [67].

The fifth category, the “other” in Table 2, had only four annotated gene sets. Their specific functions in reproductive development still require clarification.

## 4. Discussion

Inflorescence represents a highly specialized plant tissue producing seeds for propagation. Deciphering genes involved in its development is the first step to understand the essence of reproduction and of great importance for seed production manipulation. In this study, we identified 59 nonredundant wheat gene sets that were differentially expressed in wheat inflorescence and encode proteins with diverse functions. Majority of the identified genes were associated with metabolic activities and wall assembly required for the specialized process of pollen maturation and pollination, while few showed predominance or specificity in macrosporogenesis. On one hand, this could be attributed to the fact that much more ESTs from libraries made with anthers were used in the analysis, which had limitation of development stage and tissue-type coverage; on the other hand, it was probably due to the specific structure of pollen grains whose formation requires expression of a specific set of genes or gene network that made the related genes easily recognized through the differential analysis. Our results complemented well with previous studies involved in wheat floral development. The microarray-based transcriptomic analysis of anthers by Crismani et al. was mainly focused on identification of early meiotic genes [15]. In the RNA-seq data comparison of pistillody stamen versus pistil, pistillody stamen versus stamen, and pistil versus stamen, Yang et al. identified 206 genes highly correlated with stamen and pistil development [17]. Among them, however, only a few were functionally annotated as identically as the genes presented in this paper. It was noted that nearly one-third of the identified gene sets in the present study displayed differentiated expression profiles in terms of their subgenome orthologs, implying functional diversification in polyploidy wheat for the inflorescence development.

The whole process of inflorescence development is under regulatory control. A set of MADS transcription factors regulate floral organ identity specification [41, 68, 69]. The three MADS-box genes we identified, one for E-class MADS proteins and two for B-class MADS proteins, differed in their expression profiles (Figure 1), even though both *IDG001* and *IDG021* were mainly expressed in stamen, suggesting they act in concert in determining the anther and pistil identity. The pistillody in alloplasmic wheat was related to expression pattern alteration of class B genes [68]. The identification of genes related to JA, ET, and GA signaling added support for the important roles of JA, ET, and GA signaling cross-talks playing in stamen development [70, 71]. ET signaling is involved in multiple aspects of floral organ development, for instance, nectar secretion, accumulation of stigmatic exudate, and development of the self-incompatible response [72], floral organ senescing [73], pollen thermotolerance [74], and timing of anther dehiscence [71]. Cheng et al. demonstrated that GA regulates stamen development through JA signaling [75]. Mutations of genes encoding JA-biosynthetic enzymes result in failure of filament elongation, delayed anther dehiscence, and unviable pollens [70, 76, 77].

A few identified genes were associated with pollen-stigma interactions. All but *IDG028* had transcripts in spikes at meiosis and anthesis stages as well as in anthers but the abundance varied considerably. The subtilase-encoding genes related to anther dehiscence (*IDG002*) were overwhelmingly expressed in anthers, especially at the tetrad stage [60]. In male-sterile lines, their expression was downregulated [77]. Transcripts of pollen allergen-encoding *IDG035* and ACO-encoding *IDG046* were also present in spikes at this stage. Accumulating evidence indicates the involvement of ET signaling in fertilization [78, 79]. Valdiva et al. showed that disruption of a maize group-I allergen affected pollen-pollen competition for access to the ovules [80]. In addition, the galactosyltransferase-encoding *IDG006*, predominantly expressed in stamen, was related to pollen tube elongation [81].

Most of the genes showing anther-specific or predominant expression are related to tapetal and pollen developments (Table 2). Particularly worth mentioning are the gene set-encoding enzymes or proteins participating in lipid metabolism. Lipid metabolism is important to pollen development because the distinct pollen wall structure is mainly made of fatty (lipid) substances produced in the tapetum of anthers [82]. Among the lipid metabolism-related gene sets, five (*IDG017*, *IDG019*, *IDG030*, *IDG036*, and *IDG041*) encode members of 86A, 86B, and 94A subfamilies of cytochrome P450 proteins that are related to fatty acid *ω*-hydroxylation in primary fatty alcohols and suberin monomer biosynthesis for formation of anther cuticle and pollen sporopollenin in monocots and dicots [43–45, 47, 83–86]. These cytochrome P450 proteins might function in different subcellular locations, since *IDG017*, *IDG019*, and *IDG030* have secretory signal peptides, while *IDG036* and *IDG041* do not.

Pathways emerging from the lipid metabolism-related genes included those for alkane and fatty alcohol production and glycerophospholipid metabolism. Genes encoding long-chain acyl-CoA synthetase (*IDG010*) and fatty aldehyde decarbonylase (*CER1*, *IDG045*), the two enzymes involved in the plant alkane-forming pathway [87], were coexpressed in the wheat stamen. This *CER1* gene was downregulated in pistil or pistillody stamen [17], suggesting its specificity to stamen development. Very long-chain (VLC) alkanes are major components of the tryphine layer covering pollen grains and are needed for proper pollen-pistil signaling and fertility [50]. Mutation of *CER1* in both *Arabidopsis* and rice caused defective pollens [50, 51]. In *Arabidopsis*, the acyl-CoA synthetase gene *ACOS5* was upregulated in the tapetal cells. Its mutation led to failure in pollen production and pollen wall formation [88].

Fatty alcohols are components of surface lipid barriers such as anther cuticle and pollen wall [89]. Fatty acyl-CoA reductase, encoded by *IDG033* in wheat and *Ms2* in *Arabidopsis*, is the key enzyme for the production of fatty alcohols in plastids [48]. Mutation of *Ms2* led to abnormal pollen wall development and reduced pollen fertility [90, 91]. Like Ms2, the *IDG033*-encoded proteins have plastidic localization signal peptides. The encoded products of *IDG059*, *IDG032*, and *IDG037*, probably involved in the production of fatty acyl-CoA reductase substrates, all have plastidic localization signals. The diacylglycerol acyltransferase- (DGAT-) like protein encoded by *IDG057* could also carry a plastidic peptide. The *Arabidopsis DGAT1* contributes to triacylglycerol biosynthesis and its function loss causes critical defects in normal pollen and embryo development [84]. However, information about its link to the plastidial fatty alcohol pathway is still lacking. The expression profiles of these genes were different, although all expressed in stamen. *IDG059*, *IDG032*, and *IDG037* appeared to be specifically expressed in this organ.

Among the lipid metabolism-related genes, five (*IDG009*, *IDG013*, *IDG022*, *IDG027*, and *IDG054*) encode proteins putatively associated with glycerophospholipid metabolism, in agreement with the findings of Yang et al. [17]. The *IDG009*-encoded phosphoethanolamine *N*-methyltransferase (PEAMT) is the committing enzyme for choline biosynthesis. In *Arabidopsis*, silencing the PEAMT gene resulted in temperature-sensitive male sterility and salt hypersensitivity [92]; knockdown of *GPAT6*, the homolog of *IDG022,* caused defective pollen grains [46]. PEAMT and GPAT6 also affected pollen tube growth [46, 92]. Moreover, *IDG051* encode proteins predicted with lysophosphatidylethanolamine acyltransferase activities, which probably participates in the phospholipid metabolism in mitochondria.

Only 59 inflorescence development-related nonredundant gene sets were identified in this study. This could be much less than the actual number of genes differentially expressed in inflorescence tissues. We reasoned that the EST libraries used in gene mining, which had limitations in volume size and representation of tissue and developmental stages, and the strict standard used in gene mining were the main causes. A sampling probability ≤ 0.0001 has very likely increase type II error; however, it could minimize false positives, as shown in RNA-seq data analysis and RT-PCR validation, which is beneficial for correct data interpretation.

## 5. Conclusions

In this study, we identified 170 wheat genes for floral identity determination, anther and pollen development, pollen-pistil interaction, and others using the comparative transcriptomics approach. The potential importance of the identified genes to wheat inflorescence development was manifested in the enhanced or specific expression in the floral tissues. We noted that nearly one-third of the gene sets have undergone subgenome differentiation. Of the identified genes, those coding for enzymes or proteins participating in lipid metabolic pathway accounted for the largest category, implying the particularity and important roles of lipid metabolism in wheat reproductive development. This study is useful for understanding the gene network underlying wheat inflorescence morphology and fertility, which eventually will allow us to purposely manipulate fertility in breeding.

## Figures and Tables

**Table 1:** Chromosome distribution of the identified wheat inflorescence development-related genes.

Gene_ID	Chromosomes			Gene_ID	Chromosomes		
*IDG001*	1A	1B	1D	*IDG031*	*c*	*c*	3D
*IDG002*	1A	1B	1D	*IDG032*	4A	*b*	5D
*IDG003*	1A	1B	1D	*IDG033*	4A	4B	4D
*IDG004*	1A	1B	1D	*IDG034*	*c*	4B	4D
*IDG005*	1A	1B	1D	*IDG035*	5A (2)	4B	4D (2)
*IDG006*	1A	1B	1D	*IDG036*	5A	5B	5D
*IDG007*	1A	1B	1D	*IDG037*	5A	5B	5D
*IDG008*	1A	1B	1D	*IDG038*	5A	5B	5D
*IDG009*	1A	1B	1D	*IDG039*	5A	5B	5D
*IDG010*	1A	1B	1D	*IDG040*	*c*	5B (2)	5D
*IDG011*	1A	*c^1^*	*a*	*IDG041*	*b*	5B	5D
*IDG012*	*c*	1B	1D	*IDG042*	6A (8)	6B (3)	6D (3)
*IDG013*	*b*	1B	*b*	*IDG043*	*b*	6B	6D (2)
*IDG014*	2A	2B	2D	*IDG044*	6A	6B	6D
*IDG015*	2A	*a*	2D	*IDG045*	6A	6B	6D
*IDG016*	2A	2B	2D	*IDG046*	6A	*c*	*b*
*IDG017*	2A	2B	*b*	*IDG047*	6A	6B	6D
*IDG018*	*a*	2B	2D	*IDG048*	6A	6B	6D
*IDG019*	3A	3B	3D	*IDG049*	6A (2)	6B	6D
*IDG020*	3A	3B	3D	*IDG050.1*	6A	*a*	6D
*IDG021*	3A	3B	3D	*IDG050.2*	6A	*b*	6D
*IDG022*	3A	3B	3D	*IDG051*	*b*	6B	6D
*IDG023*	3A	3B	3D	*IDG052*	*c*	6B (2)	*c*
*IDG024*	3A	3B	3D	*IDG053*	*a*	6B	*a*
*IDG025*	3A	3B	3D	*IDG054*	7A	7B	7D
*IDG026*	3A	3B	3D	*IDG055*	7A	7B	7D
*IDG027*	3A	*c*	3D	*IDG056*	7A	7B	7D
*IDG022*	3A	3B	3D	*IDG057*	7A	*a*	7D
*IDG029*	3A (2)	3B (2)	3D (2)	*IDG058*	*b*	7B	*b*
*IDG030*	3A	3B	3D	*IDG059*	*c*	*c*	7D

*a*: corresponding gene was not found; *b*: genomic DNA is available but not supported by ESTs; *c*: the gene was not inflorescence predominantly expressed. Copy number is shown in parenthesis.

**Table 2:** Functional classifications of the annotated gene sets.

Gene set	Annotation	Molecular or biological function	Reference
1. Allergen			
*IDG035*	Group 3 grass pollen allergen		[93]
*IDG039*	Peamaclein-like		
2. Expression regulation			
*IDG001*	MADS-box transcription factor WM14	Organ identity specification	[68]
*IDG003*	Gibberellin-regulated protein II-like	Gibberellin signaling	[70]
*IDG005*	Cysteine protease-like protein	Ethylene signaling	[73]
*IDG011*	Histone H2A	Chromosome modeling	
*IDG012*	Histone H2A	Chromosome modeling	
*IDG021*	MADS-box transcription factor	Organ identity specification	[68]
*IDG031*	Glyoxysomal fatty acid beta-oxidation multifunctional protein MFP-a	JA signaling	[94]
*IDG044*	Transcription factor AGL6-like	Organ identity specification	[69]
*IDG046*	1-aminocyclopropane-1-carboxylate oxidase, EC 1.14.17.4	Ethylene signaling	[72, 95]
*IDG048*	Histone H2B	Chromosome modeling	
*IDG052*	1-aminocyclopropane-1-carboxylate oxidase, EC 1.14.17.4	Ethylene signaling	[72, 95]
3. Anther development and pollen wall formation			
*IDG004*	Anther-specific protein RTS-like	Tapetal development	[56]
*IDG006*	Galactosyltransferase, EC 2.4.1.-	Cell wall assembly	[81]
*IDG007*	Protease inhibitor/seed storage/LTP family protein-like	Pollen wall formation	[53]
*IDG009*	Phosphoethanolamine *N*-methyltransferase EC 2.1.1.103	Lipid metabolism	[92]
*IDG010*	Long chain acyl-CoA synthetase, EC 6.2.1.3	Lipid metabolism	[92]
*IDG013*	Triacylglycerol lipase, EC 3.1.1.3	Lipid metabolism	
*IDG017*	Cytochrome P450 86B1-like, EC 1.14.15.-	Lipid metabolism	[83]
*IDG018*	Putative RAFTIN1/BURP domain-containing protein	Pollen wall formation	[53]
*IDG019*	Cytochrome P450 94A1-like	Lipid metabolism	[45]
*IDG020*	Late cornified envelope-like proline-rich protein	ROS detoxification	[59]
*IDG022*	Glycerol-3-phosphate acyltransferase 6, EC 2.3.1.198	Lipid metabolism	[46]
*IDG025*	Anther-specific protein RTS-like	Tapetal development	[56]
*IDG027*	Nonspecific phospholipase C2, EC 3.1.4.3	Lipid metabolism	
*IDG030*	Cytochrome P450 86A2-like	Lipid metabolism	[83]
*IDG032*	Acyl-[acyl-carrier-protein] desaturase, EC 1.14.19.2	Lipid metabolism	
*IDG033*	Fatty acyl-CoA reductase 2. EC 1.2.1.42	Lipid metabolism	[48]
*IDG036*	Cytochrome P450 86B1, fatty acid *ω*-hydroxylase, EC 1.14.15.3	Lipid metabolism	[83]
*IDG037*	Acyl-[acyl-carrier-protein] desaturase, EC 1.14.19.2	Lipid metabolism	
*IDG038*	Acidic peroxidase, EC 1.11.1.7	Phenylpropanoid biosynthesis	[52]
*IDG041*	Cytochrome P450 86A1-like, fatty acid *ω*-hydroxylase	Lipid metabolism	[83]
*IDG045*	Fatty aldehyde decarbonylase, EC 4.1.99.5	Lipid metabolism	[50, 51]
*IDG050*	Nonspecific lipid-transfer protein C6-like	Pollen wall formation	[53]
*IDG051*	Acyltransferase-like protein, EC 2.3.1.-	Lipid metabolism	
*IDG054*	Triacylglycerol lipase 2-like, EC 3.1.1.13	Lipid metabolism	
*IDG055*	Bidirectional sugar cr SWEET	Pollen wall formation	[54, 55]
*IDG056*	RAFTIN1 protein/BURP domain protein-like	Pollen wall formation	[53]
*IDG057*	Acyltransferase-like protein, EC 2.3.1.-	Lipid metabolism	
*IDG059*	Acyl carrier protein	Lipid metabolism	
4. Pollination and pollen-stigma interactions			
*IDG002*	Subtilisin-like protease	Anther dehiscence	[60]
*IDG008*	Chalcone synthase-like	Biosynthesis of flavonols	[61]
*IDG028*	Protodermal factor 1-like	ROS homeostasis	[64]
*IDG040*	Chemocyanin	Pollen tube attraction	[62]
5. Others			
*IDG014*	Nitrate-induced NOI protein	Plant defense	[96]
*IDG034*	Nucleoside diphosphate kinase, EC 2.7.4.6	Nucleotide triphosphate generation	
*IDG053*	Zinc transporter-like	Early reproductive development	
*IDG058*	Heat shock protein	Thermotolerance	

## Supplementary Materials

Supplementary 1 Figure S1: expression of a selected set of identified genes in different tissues, examined using qRT-PCR. The expression levels were estimated relative to that in stamen. The tissues used included kernel 9th day postanthesis, root, node, internode, flag leaf, glume, lemma, palea, lodicule, stamen, pistil, and rachis at the heading stage of common wheat landrace “Wangshuibai.”

Supplementary 2 Table S1: cDNA libraries used in this study.

Supplementary 3 Table S2: primers used in RT-PCR.

Supplementary 4 Table S3: sequence, annotation, and expression specificity of 170 identified genes. ^∗^ and ^∗∗^ significantly different at *P* = 0.05 and 0.01, respectively.
