# Supplementary figures and images for: Identification of Wheat Inflorescence Development-Related Genes Using a Comparative Transcriptomics Approach
Source: Int J Genomics. 2018 Feb 8;2018:6897032. doi: 10.1155/2018/6897032 (PMC5822904; doi:10.1155/2018/6897032)

IDG003-1A/1B/1D

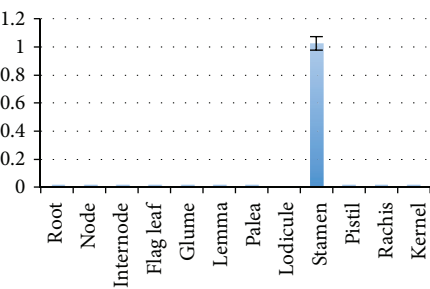

IDG004-1A/1B/1D

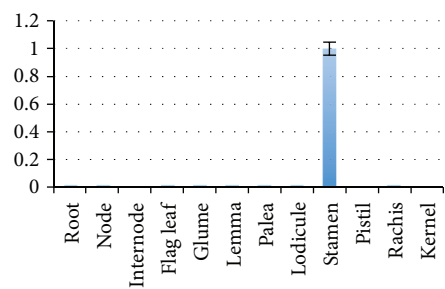

IDG006-1A/1B/1D

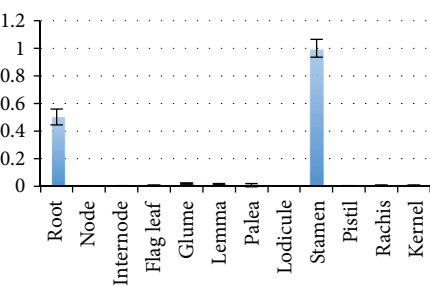

IDG007-1A/1B/1D

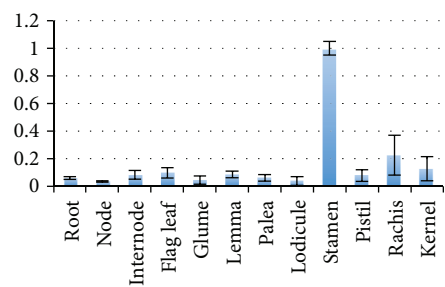

IDG008-1A/1B/1D

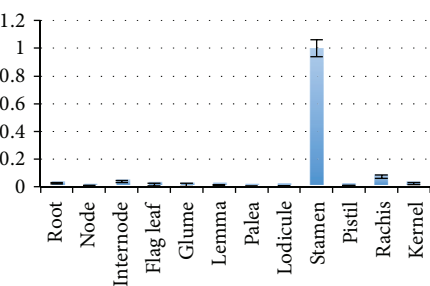

IDG015-2A/2D

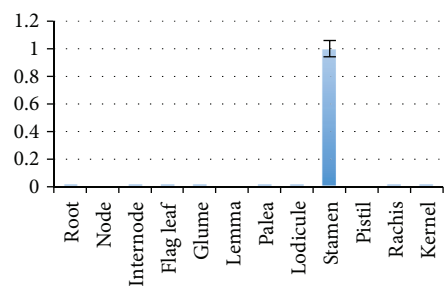

IDG018-2B/2D

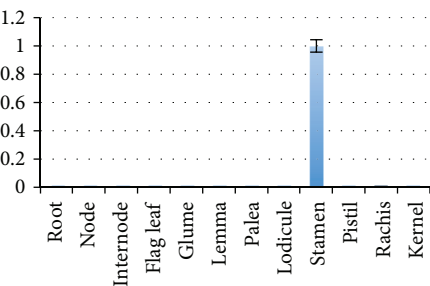

IDG021-3A/3B/3D

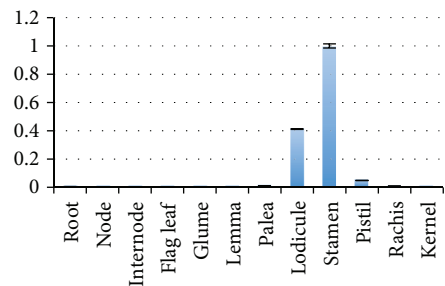

IDG024-3D

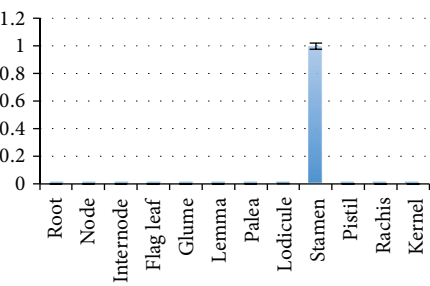

IDG030-3A/3B/3D

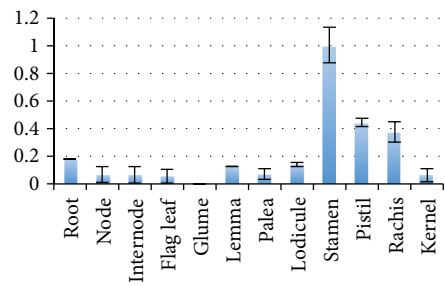

IDG042.1-6D

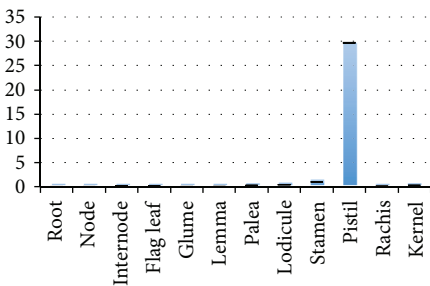

IDG043-6B/6D

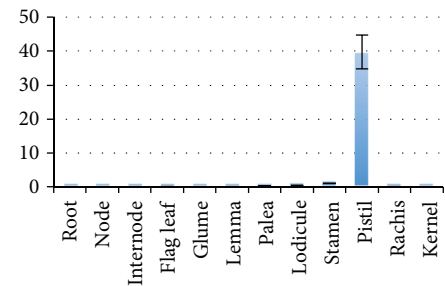

Supplement: Supplementary 1 — Figure S1: expression of a selected set of identified genes in different tissues, examined using qRT-PCR. The expression levels were estimated relative to that in stamen. The tissues used included kernel 9th day postanthesis, root, node, internode, flag leaf, glume, lemma, palea, lodicule, stamen, pistil, and rachis at the heading stage of common wheat landrace “Wangshuibai.” [file 6897032.f1.pdf]
