# Supplementary material for: Identification of Wheat Inflorescence Development-Related Genes Using a Comparative Transcriptomics Approach
Source: Int J Genomics. 2018 Feb 8;2018:6897032. doi: 10.1155/2018/6897032 (PMC5822904; doi:10.1155/2018/6897032)
Supplement: Supplementary 2 — Table S1: cDNA libraries used in this study. [file 6897032.f2.docx]

**Table S1.** cDNA libraries used in this study

| **Library Name** | **ESTs** | **Organ** | **Develop. stage** |
| --- | --- | --- | --- |
| Wheat unstressed root cDNA library | 1025 | root | full tillering |
| ITEC MWL Wheat Root Library | 1038 | root | 8 day old |
| AZO4 | 2513 | root |  |
| Wheat etiolated seedling root cDNA library | 4107 | root | five day old etiolated seedling |
| Y.Ogihara unpublished cDNA library Wh_RDr | 10635 | root | root of desiccated seed |
| Y. Ogihara unpublished cDNA library, Wh_r | 5924 | root | Feekes' scale 1 |
| AZO2 | 8025 | root |  |
| Y. Ogihara unpublished cDNA library, whatl | 12858 | root |  |
| Y. Ogihara unpublished cDNA library, whsct | 13649 | root | Y. Ogihara unpublished cDNA library, whsct |
| Y. Ogihara unpublished cDNA library, whatlal | 13750 | root |  |
| wr1 | 14967 | root | 7 day old seedling, light grown |
| root_Halberd_unknown | 38446 | root |  |
| Y.Ogihara unpublished cDNA library Wh_KMP | 11354 | stem | shoot grown under continuous light |
| Wheat 4-day-old etiolated seedling shoot library | 4128 | stem | 4-day-old etiolated seedling |
| Wheat unstressed seedling shoot cDNA library | 2250 | stem | Etiolated shoot, five day old seedling |
| F1 | 4653 | leaf | leaf one |
| wlm0 | 4551 | leaf | seedlings 0 hr after inoculation with Erysiphe graminis f. sp tritici |
| AZO3 | 3300 | leaf |  |
| AZO1 | 2881 | leaf |  |
| Y.Ogihara unpublished cDNA library Wh_HGCPCDAM | 9008 | flower | anther at meiosis |
| Y.Ogihara unpublished cDNA library Wh_PCDAM | 9205 | flower | anther at meiosis |
| Y.Ogihara unpublished cDNA library Wh_GCPCDAM | 9424 | flower | anther at meiosis |
| Wheat meiotic anther cDNA library | 9139 | flower | anther, meiotic stages pre-meiosis-metaphase I |
| wpa1c | 4858 | flower | anther, pre-meiotic anthers JIC |
| waw1c | 1475 | flower | anthers, meiosis |
| wdi1c | 2201 | flower | developing inflorescence +/- 4 cm |
| Y. Ogihara unpublished cDNA library, Wh_oh | 5874 | flower | Feekes' scale 10.5, pistil at heading date |
| Y. Ogihara unpublished cDNA library, Wh_h | 6274 | flower | Feekes' scale 10.5, spike at heading date |
| Y. Ogihara unpublished cDNA library, Wh_f | 6948 | flower | Feekes' scale 10.5.1, spike at flowering date |
| Y. Ogihara unpublished cDNA library, Wh_yf | 6307 | flower | Feekes' scale 6, spikelet at early flowering |
| Y. Ogihara unpublished cDNA library, Wh_yd | 6697 | flower | Feekes' scale 6, spikelet at late flowering |
| Y. Ogihara unpublished cDNA library, Wh | 8086 | flower | Feekes' scale 9, spike at meiosis |
| wlp1c | 2306 | flower | lemma and palea |
| OV | 5366 | flower | ovary |
| wip1c | 1955 | flower | pistils |
| Wheat pre-anthesis spike cDNA library | 11197 | flower | spike before anthesis, adult plant |
| Y.Ogihara unpublished cDNA library Wh_FL | 24568 | flower | spikelet, early flowering |
| ITEC MUG Wheat Spikelet Library | 2123 | flower | spikelets, Feekes' scale 6-7 |
| Wheat meiotic floret cDNA library | 1501 | flower | whole florets with anthers, meiotic stages pre-meiosis-metaphase I |
| Y. Ogihara unpublished cDNA library, Wh_SL | 6920 | seed | Feekes' scale 11.3, seed DPA30 |
| Y.Ogihara unpublished cDNA library Wh_DPA20 | 11308 | seed | seed DPA20 |
| Y. Ogihara unpublished cDNA library, Wh_e | 6424 | seed | Feekes' scale 11.2, seed DPA10 |
| Y.Ogihara unpublished cDNA library Wh_MS | 10729 | seed | seed DPA5 |
| Y.Ogihara unpublished cDNA library Wh_OKCS | 10461 | seed | seed DPA5 |
| G118 | 8172 | seed | grain (118 degrees per day after pollination) |
| GR45 | 6921 | seed | grain (45 degrees per day after pollination) |
| Wheat_EST_endosperm_library | 2564 | seed | developing_endosperm_tissue_6_8_10_dpa |
| wheat EST endosperm library | 4433 | seed | developing endosperm tissue 8, 10 and 12 DPA |
| G550 | 4810 | seed | grain (550 degrees per day after pollination) |
| TaE05 | 5881 | seed | developing seeds, 5 days after anthesis |
| G468 | 4646 | seed | grain (468 degrees per day after pollination) |
| TaE15 | 5496 | seed | developing seeds, 15 days after anthesis |
| G608 | 4839 | seed | grain (608 degrees per day after pollination) |
| G174 | 4602 | seed | grain (174 degrees per day after pollination) |
| wdk3c | 5054 | seed | kernel, 14 days after anthesis |
| wdk2c | 5017 | seed | kernel, 7 days after anthesis |
| G750 | 7483 | seed | grain (750 degrees per day after pollination) |
| wdk1c | 4860 | seed | kernel, 3 days after anthesis |
| G356 | 3595 | seed | grain (356 degrees per day after pollination) |
| Wheat developing grains cDNA library | 3649 | seed | whole grains, 3-44 days post anthesis seed |
| Wheat dormant embryo cDNA library | 2954 | seed | mature seed, seed embryo |
| Wheat endosperm cDNA library | 2824 | seed | endosperm, 5 to 30 days post anthesis seed |
| ITEC WHE Wheat Endosperm Library | 2022 | seed | endosperm, 5-30 days post anthesis |
| Cheyenne wheat endosperm cDNA library | 1216 | seed | 5-30 days post anthesis seed |
| ITEC SCU Wheat Endosperm Library | 1152 | seed | endosperm |
| ITEC CSB Wheat Endosperm Library | 1047 | seed | endosperm, 8-12 days post anthesis |
| N:130 | 1013 | seed | embryo, 2 days post germination |
