# Supplementary material for: Identification of Wheat Inflorescence Development-Related Genes Using a Comparative Transcriptomics Approach
Source: Int J Genomics. 2018 Feb 8;2018:6897032. doi: 10.1155/2018/6897032 (PMC5822904; doi:10.1155/2018/6897032)
Supplement: Supplementary 3 — Table S2: primers used in RT-PCR. [file 6897032.f3.docx]

**Table S2** Primers used in RT-PCR

| **Primer ID** | **Examined Genes** | **Forward Primer (5’- 3’)** | **Reverse Primer (5’- 3’)** |
| --- | --- | --- | --- |
| WGRA0088 | *IDG002-1B/1D* | AAGACTATCACGGGGAACAGCAG | CACCTTCACCTTGAGCCACTTG |
| WGRA0104 | *IDG003-1A/1B/1D* | GCAAAGCTAGGGTGCTCATGTG | TTGTGCGTGGTCATGTTGGC |
| WGRA0083 | *IDG004-1A/1B/1D* | TGAACGGGATGATGCAGTGC | GTGTTTTGACGGGCGTATGG |
| WGRA0105 | *IDG006-1A/1B/1D* | GGGATGCTGACGAACAGGC | GTTGATGGAGATGTAGGAGGCG |
| WGRA0092 | *IDG007-1A/1B/1D* | GCGATGCTCCTCCTCCTTGC | CAGTTGCCAGGGATGCTGAC |
| WGRA0099 | *IDG008-1A/1B/1D* | GACCTACCTCCAGGACACCAGC | CCGTGGCGTCAATGTTGG |
| WGRA0077 | *IDG015-2A/2D* | TTCTGCTTCTGCTGCTCGTAGC | AAGCTCCCACAATCATCCGC |
| WGRA0086 | *IDG016-2A* | AATCACACACTCCACCGATGGCAC | TTGTCGTCGTCGTCCTTGTTGTCC |
| WGRA0080 | *IDG018-2B/2D* | ATGTAACCCACGGCTCCAAGCAA | TGGCAGTAAAAGACCTCCACAGG |
| WGRA0084 | *IDG020-3A/3B/3D* | GGATTGCTACAAGGCATGTGGG | ATTAAGTGGCTTTCGGCGGG |
| WGRA0103 | *IDG021-3A/3B/3D* | AGAACCAGAGCAACCGCCAG | CGCCTTTCAAGTGCCTGAGC |
| MAG6170 | *IDG024-3D* | AACTACGCCTGCGACAAGACCTGC | ACGCACGCACCGCATCATCTGGAT |
| WGRA0079 | *IDG025-3B* | GCTGGTGGCTACGAGATGAATG | TGGATTGATTGGGGATCAGAGC |
| WGRA0100 | *IDG026-3A/3B/3D* | CGACTTCTGATCGACGACCCTC | CACGCACTGCATCTGCACATC |
| WGRA0095 | *IDG029.1-3A/3B* | TCTCCTCTCCTCGTGCGACG | GGCAGCAGCAGGTCTTTTTGTC |
| WGRA0094 | *IDG029.1-3D* | CCTTCTCCTCTCCTCGTGCG | GGCAGCAGCAGGTTTTTTGTC |
| WGRA0081 | *IDG029.2-3A/3B/3D* | CCGCAAAGTGAAGGATAAGCC | CAGCAACAGTGGGTTTTGTCG |
| WGRA0096 | *IDG030-3A/3B/3D* | ACCCAAAAGGCAGTGAGTTCG | CAGACAAGCAGTCAAGGTCAGTTG |
| WGRA0098 | *IDG035-5A/4B/4D* | GGAGAAGGGGTCCGACAAAAAG | CCAGTGGCTTGGAGCACGAG |
| WGRA0106 | *IDG037-5A/5B/5D* | ATGACTACTTGGTCTGTCTGGTTGG | CTGTATGGTCTTCTCAATCTGCCTC |
| WGRA0089 | *IDG038-5B/5D* | CATCAACTCGCTCAGGGGTTTC | CGTTGGTGAACTTCTGCACGAG |
| WGRA0101 | *IDG042.1-6D* | TTCCCCGCTCTGGTCCTTC | CAGCACCGTTGTATTCTCCAATG |
| WGRA0102 | *IDG043-6B/6D* | ATCGGCAGCAGCAGGTGTC | TGGCGAGGAGGAAGAGACG |
| WGRA0085 | *IDG047-6A/6D* | AAGGTGGCAACAAAGGTGACG | GGCTGGTTGTGGAAGCATTTG |
| WGRA0091 | *IDG050.1-6A* | TGCGGCGAAAAAGAGCATC | TAGGGTGCGGTTGAAGATGTGG |
| WGRA0093 | *IDG053-6B* | GCATAGTTCAGGCAAAGTTCAAGG | CTTCGGGTTGGTAAGTTCCTCAG |
| WGRA0087 | *IDG054-7B/7D* | GAAGCATTACAAGCAACCACGC | TGAGCATAGTCGGGCAAGTATTG |
| WGRA0078 | *IDG056-7A/7B* | CCGATGATGAACCGAGCAAGAG | AAAGAACACCGTCGTCGTCTCC |
| MAG5725 | *α-tubulin* | ATCTCCAACTCCACCAGTGTCG | TCATCGCCCTCATCACCGTC |
